# Study protocol for a group-based motivational interviewing brief intervention to reduce substance use and sexual risk behavior among young adults experiencing homelessness

**DOI:** 10.1186/s13722-020-00201-x

**Published:** 2020-07-28

**Authors:** Joan S. Tucker, Elizabeth J. D’Amico, Eric R. Pedersen, Anthony Rodriguez, Rick Garvey

**Affiliations:** 1 RAND Corporation, 1776 Main Street, PO Box 2136, Santa Monica, CA 90407-2138 United States; 2 Department of Psychiatry and Behavioral Sciences, Keck School of Medicine, University of Southern California, 250 Alcazar Street, Suite 2200, Los Angeles, CA 90033 United States; 3 RAND Corporation, 20 Park Plaza, Suite 920, Boston, MA 02116 United States

**Keywords:** Substance use, Unprotected sex, Intervention, Motivational interviewing, Homeless, Young adults

## Abstract

**Background:**

Young people experiencing homelessness have alarmingly high rates of alcohol and other drug (AOD) use, which is associated with sexual risk behaviors such as unprotected sex, trading sex, and sex with multiple casual partners. Few risk reduction programs for this population have been developed and rigorously evaluated, particularly those that address both of these interrelated behaviors, use a collaborative and non-judgmental approach, and are feasible to deliver in settings where homeless young people seek services. This paper describes the protocol of a study evaluating a four-session Motivational Interviewing (MI)-based group risk reduction intervention for this population. The protocol has been shown to be efficacious in pilot work over 3 months with 200 homeless young adults [1]. The current study seeks to refine the intervention protocol and evaluate the program on a larger scale.

**Methods/design:**

In a cluster-cross-over randomized controlled trial, 18–25 year olds will receive the AWARE risk reduction program (n = 200) or standard care (n = 200) at one of three drop-in centers serving homeless youth in the Los Angeles area. We will evaluate intervention effects on primary outcomes of AOD use and sexual risk behavior, as well as secondary outcomes of health-related quality of life and social stability, over a 12-month period.

**Discussion:**

This project has the potential to fill a significant gap in prevention services by demonstrating that a brief intervention, feasible to deliver within settings where young people experiencing homelessness typically seeks services, can significantly reduce the interrelated problems of AOD use and sexual risk behavior.

*Trial registration* ClinicalTrials.gov Identifier: NCT03735784. Registered November 18, 2018, https://clinicaltrials.gov/ct2/show/record/NCT03735784 (retrospectively registered)

## Background

Unaccompanied young people (under age 25) experiencing homelessness, the focus of this study, constitute a large and vulnerable segment of the population in the United States. The most recent point-in-time homeless count indicates that there are 35,038 people under the age of 25 who are homeless and not accompanied by a parent or guardian on any given night [2]. Los Angeles County, the geographic focus of the study described here, has one of the largest populations of young experiencing homelessness in the U.S. [2].

## Young people experiencing homelessness have high rates of sexual risk behavior and related consequences

Engagement in sexual risk behavior such as multiple partnerships, sexual concurrency, sex under the influence of drugs or alcohol, and trading sex is common among young people experiencing homelessness [3–8], which can contribute to their relatively high rates of HIV and sexually transmitted diseases (STDs) [9, 10]. In fact, young people experiencing homelessness are estimated to be 6–12 times more likely than other youth to become infected with a human immunodeficiency virus (HIV) [11], and 1 in 5 homeless youth report having an STD [12]. Pregnancy is common as well, with national data finding that 44% of 18 to 25 year old women experiencing homelessness report being pregnant or a parent [13]. In a probability sample of 113 young females experiencing homelessness in Los Angeles County, 28% report that they had been pregnant or tried to become pregnant in the past 3 months [14]. Condom use can be an effective means of protection against both HIV/STDs and pregnancy, yet 40–70% of young people experiencing homelessness report engaging in unprotected sexual intercourse [4, 7, 15, 16].

## Substance use is common among young people experiencing homelessness as well, and is often associated with sexual risk behavior

High rates of AOD use [3, 17–21], including injection drug use [22, 23], are consistently found in studies of youth experiencing homelessness. For example, in a survey of 419 homeless youth in Los Angeles County, past month AOD use was reported by 68% for alcohol, 66% for cannabis, 15–16% for methamphetamine and ecstasy, and 9% for injection drugs [19, 24]. An estimated 60%–70% of homeless youth meet lifetime criteria for a substance use disorder [25–28]. Thus, it is not surprising that many homeless youth engage in sex while high or intoxicated [16, 29]. AOD use is also a risk factor for HIV/STD transmission through its association with high-risk sexual behavior such as unprotected sex, sex with a partner who injects drugs or is HIV-positive, and trading sex [16, 30–33].

## Brief, yet effective AOD and sexual risk reduction programs for young people experiencing homelessness are needed [34]

To date, brief interventions for these young people have tended to focus solely on AOD or HIV/STD prevention alone (i.e., they have not targeted the unique combined risks from both AOD use and risky sex behaviors evident among this population, such as impaired decisions to have condomless sex following heavy drinking or drug use), and have demonstrated minimal long-term efficacy. One study evaluated a single-session one-on-one Motivational Interviewing (MI) intervention, which presented youth with personalized feedback about their patterns of risk associated with AOD use [35]. The intervention group showed reduced illicit drug use other than cannabis at 1-month follow-up compared to the control group; however, this effect was not sustained by 3-month follow-up, and no effects were found on alcohol or cannabis use. Another study evaluated a similar type of one-on-one motivational intervention, but delivered over four sessions with an average total treatment exposure of 73 min [36]. The program did not have a statistically significant effect on AOD use at 3-month follow-up. Other brief interventions include a street outreach HIV prevention intervention that offered service listings/referrals and supplies (e.g., condoms and bleach), which did not find a statistically significant effect on condom use [37], and a peer-delivered HIV prevention intervention, which did not significantly reduce AOD use or sexual risk behavior at 3-month follow-up [38]. Finally, a 2-session HIV intervention for homeless youth receiving substance use treatment through a drop-in center had a statistically significant effect on condom use at 6-month follow-up, but this effect dissipated by 12 months [39]. Although this research has demonstrated the feasibility of implementing risk reduction interventions with homeless youth, very brief motivational interventions have not demonstrated long-term efficacy compared to standard care, which typically consists of case management, basic services (food, clothing), and other programs to meet the therapeutic, health, and other needs of homeless youth. A review of risk reduction interventions for runaway youth and those experiencing homelessness concluded that programs need to be intensive enough to address the multiple and interrelated risk behaviors that most homeless adolescents and young adults exhibit, yet practical to deliver in the busy service settings where these young people routinely seek services [40]. Of course, youth with a substance use disorder may need more intensive treatment than a brief intervention is designed to provide.

## Preliminary findings of AWARE

We developed AWARE to address this important gap in prevention services for young adults experiencing homelessness. AWARE is a four-session MI-based group risk reduction intervention that focuses on both substance use and sexual risk behavior. The pilot evaluation of AWARE involved 200 homeless 18–25 year olds (mean age = 21.8) who were recruited from two drop-in centers in Los Angeles County [1, 78]. The sample was 73% male, 79% self-identified straight/heterosexual, and racially/ethnically diverse (31% non-Hispanic White, 25% African American, 24% Hispanic, 21% multiracial/other). Retention in the AWARE program was excellent with 79% of participants attending multiple sessions, and participants reported high levels of satisfaction with the program [79]. Surveys were completed at baseline and three months after program completion. We were able to achieve a 91% follow-up rate by using a range of methods for tracking and retention the sample [80], which will also be used in this larger trial.

For past 30 day alcohol use, we used information on quantity and frequency of alcohol use to categorize participants as non-drinkers, non-heavy drinkers (always < 5 drinks per day), heavy drinkers (5 + drinks on 1 to 4 occasions), and frequent-heavy drinkers (5 + drinks on 5 or more occasions). We then used multinomial logistic regression to compare each drinking group to the non-drinking group. Results indicated that AWARE participants had a lower likelihood of being a frequent-heavy drinker (vs. a non-drinker) compared to control participants at 3-months follow-up (*p *= .05; Cohen’s *d* = 0.22). In addition, we assessed frequency of alcohol use in the past 3 months (0 = *never* to 7 = *everyday*), finding that AWARE participants reported lower frequency of alcohol use at follow-up compared to control participants (*p* = .01; *d *= 0.31).

While we did not find statistically significant intervention effects on drug use, we did find effects on motivation to reduce/quit their use of drugs other than marijuana. Items were rated on a scale from 0 = *not at all* to 10 = *extremely*, with AWARE participants showing a significant positive difference in their readiness to reduce/quit their use (*p* = .02; *d* = 0.32) and confidence in their ability to reduce/quit their use (*p* = .02, *d* = 0.36), as well as a marginally significant positive difference in the importance of reducing/quitting their use (*p* = .09, *d* = 0.24), compared to control participants. Limitations in our assessment of drug use for the pilot study likely contributed to weaker intervention effects for drug use behavior compared to motivation to change drug use. Note that the curriculum does not include specific references to marijuana (although it is discussed if someone brings it up during a session), due to our formative work which indicated that 18–25 year olds experiencing homelessness did not want to attend a program that addressed marijuana use.

In addition to these promising results for AOD outcomes, we found that AWARE participants showed a greater increase in their condom use self-efficacy compared to control participants at follow-up (*p* = 0.05, *d* = 0.27). For unprotected sex, we did not find a significant intervention effect in the full sample that included sexually inactive participants. However, we conducted a secondary analysis on a subsample that reported having multiple (2 or more) partners in the past 3 months at both assessments. AWARE participants showed a larger and significant reduction in the proportion of unprotected sexual events (baseline: *M* = 0.45, *SD* = 0.44; follow-up: *M* = 0.24, *SD* = 0.35, *t* = 2.15, *p* = .04, *d* = 0.60); there was no change among control participants (baseline: *M* = 0.46, *SD* = 0.44, follow-up: *M* = 0.49, *SD* = 0.48).

## The present study

The present study is designed to evaluate the long-term effects of AWARE. Its primary aim is to investigate whether 18–25 year olds experiencing homelessness who participate in AWARE show reductions in AOD use and sexual risk behavior outcomes over a 12-month period compared to a Usual Care control sample who do not receive the program. This builds on the pilot study by expanding outcome assessment from 3 months to 12 months, including a third drop-in center site and enrolling a larger sample, and looking at secondary outcomes beyond AOD use and sexual risk behavior. A secondary aim focuses on determining whether AWARE participation is associated with improvements in health-related quality of life (mental, physical, social) and social stability (e.g., education, employment, income, housing). More intensive interventions with young people experiencing homelessness have shown positive effects on these types secondary outcomes [43]; thus, it is of interest to examine whether a brief AOD intervention such as AWARE may have similar effects.

In the protocol below, we focus on the primary (AOD and sexual behavior) and secondary (health related quality of life, social stability) aims of the project. We first describe the eligibility criteria for study participants and our recruitment approach, as well as the primary and secondary outcome measures. The format and key components of the intervention are also described. Finally, we describe the analytic plan for the primary and secondary aims.

## Methods/design

### Participants

The intervention is targeted towards young adults experiencing homelessness. Thus, eligibility criteria include: 1. being between the ages of 18 and 25; 2. seeking services (e.g., food, clothing) at one of the participating drop-in centers serving young people experiencing homelessness in Los Angeles County; 3. no cognitive impairment observed during the screening process; 4. planning to be in the study area for the next month; and 5. English speaking. We are focusing specifically on 18–25 year olds in this study for three reasons: (a) the vast majority (85%) of the unaccompanied homeless youth population is in this age range; (b) a wider age range among participants may adversely affect group cohesion and dynamics in this group-based program; and (c) important developmental differences between adolescents (12–17) and young adults would require tailoring of the program curriculum and conducting separate groups, which is beyond the scope of this study. AWARE can be used as both prevention and intervention; as such there are no eligibility criteria based on substance use severity. Although there is less compelling evidence that brief MI is effective with people who have severe substance use disorders, we do not want to withhold a potentially beneficial treatment from anyone who is interested in the program. We also want to provide our program to those who are interested even if they do not fit a threshold for heavy AOD use. Fortunately, our pilot results suggest that the AWARE program is appropriate and beneficial for homeless young adults in general, and not just those who are at relatively low risk (based on GAIN-SS scores in the case of substance use outcomes, and number of sex partners in the case of sexual behavior outcomes) [1]. We plan to enroll 400 participants at baseline, who are expected to represent the demographics of the broader population of 18–25 year olds experiencing homelessness in Los Angeles County. All study materials and procedures will be approved by RAND’s Human Subjects Protection Committee and a Certificate of Confidentiality from the National Institutes of Health will protect data from subpoena.

### Procedures

This study is using a form of group-randomized design [44, 45], with crossover of conditions and groups to avoid problems of power reduction associated with conventional group randomization [46]. The unit of analysis is the individual, but individuals are assigned to groups based on the agency where they are seeking services. Specifically, young adults at three drop-in centers serving young people experiencing homelessness are either in the intervention condition (*N* = 200) or control condition (*N* = 200). Drop-in centers are typically a first stop resource for these youth to address their basic needs for food and hygiene, and a place where they can go during the day to receive services, or get connected with services outside the drop-in centers, to address their higher-level needs [41]. Drop-in centers typically try to break down barriers and take a “come as you are” approach to engaging youth in available services [42]. Participants in the intervention condition receive the AWARE program, whereas participants in the Usual Care control condition have access to the full range of programs and services that are already available at the drop-in center (these programs and services are available to intervention participants as well). In addition, all participants receive an HIV informational brochure that discusses the connection between AOD use and HIV risk, and a Community Resource Guide that lists free or low-cost AOD and HIV-related services in the study area.

**Fig. 1:**
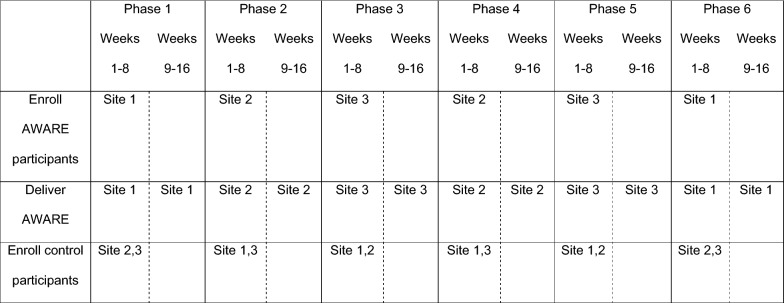
Example of cluster cross-over design used in AWARE evaluation

**Fig. 2:**
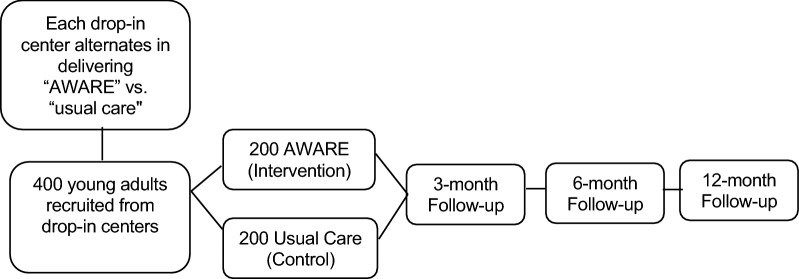
Randomized controlled trial study flow

**Fig. 3:**
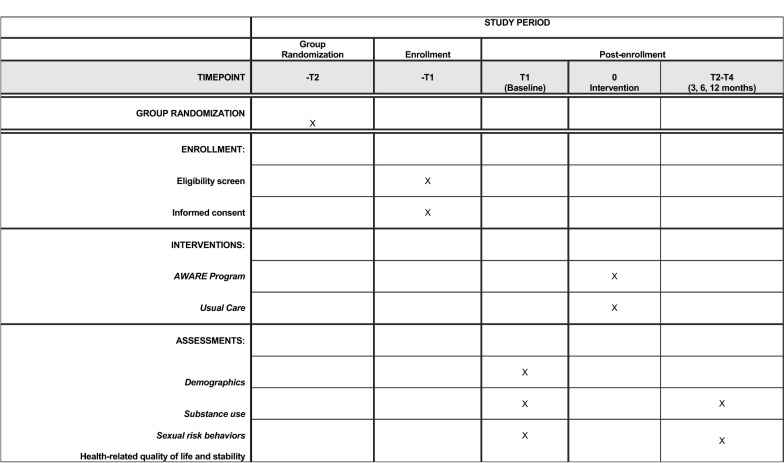
SPIRIT flow diagram of the RCT schedule of enrollment, interventions, and assessments

Recruitment occurs in a series of 16-week cycles, with the three drop-in center sites alternating across cycles in serving as the intervention site or control site (see Fig. 1). Recruitment is done by advertising the study at the drop-in centers, and through soliciting volunteers during recruitment visits. Individuals sign up on a sign-in sheet each day to participate in the study. Depending upon the number who sign up, they are randomly selected from the pool of interested individuals and screened for eligibility. Eligible participants provide written consent and complete a baseline scannable self-report survey, which takes approximately 30 min, and is completed in the presence of a staff member who can provide assistance if needed. Participants in both conditions are recruited during the first 8 weeks of each cycle, until our target enrollment number for that cycle is reached, which leaves the remainder of the cycle as a “wash out” period to reduce the likelihood of contamination across conditions. The four-session AWARE program is delivered four times during each 16-week cycle, allowing intervention participants multiple opportunities to complete all sessions. Figure 2 depicts participant flow through the study, and Fig. 3 contains a SPIRIT (Standard Protocol Items: Recommendations for Interventional Trials) flow diagram of the RCT schedule of enrollment, interventions, and assessments.

### Description of the intervention

Each of the four AWARE sessions lasts approximately 45 min. Although some sessions focus more heavily on sexual risk and others on AOD use, each session emphasizes the interrelated nature of these risk behaviors (see Table 1). This interactive program is designed to be delivered in a small group setting (i.e., fewer than 9–10 members). It is also designed to have a “rolling admission” so that participants can enter the program at any of the four sessions. This is an important feature in that someone can access the program without delay when they are motivated to do so, and is a common way to reach young people in these types of settings [47–49]. AWARE participants receive a small ($5) incentive for each session that they attend. AWARE is designed to be facilitated by interventionists who have received training on both the curriculum and the use of motivational interviewing, and have demonstrated proficiency in delivering the sessions. As with other MI-based interventions developed by our team members (e.g., [48, 50, 54, 55]), it is not necessary for facilitators to have a specific college degree or education background to fill this role. However, AWARE facilitators should be knowledgeable, enthusiastic, and comfortable working with this population.

**Table 1:** Program overview

Session	Goals
1	***HIV/STIs: knowing the facts*** Provide basic information on HIV/STIs through provision of personalized feedback. Discuss substance use as a risk factor for HIV/STI transmission Discuss prevalence of HIV/STIs and why it might be higher among young adults who are homeless Discuss how substance use puts people at higher risk for HIV/STIs Help participants better evaluate partner risk in terms of HIV/STI transmission Provide normative feedback about HIV-related behaviors Help participants develop skills to effectively use a condom
2	***HIV/STIs: dealing with risky situations*** Help participants identify people, places, situations, and feelings that may trigger their sexual risk behaviors (emphasizing the role of substance use as a trigger for sexual risk behavior). Discuss ways to avoid triggers Help participants strategize on how to deal with triggers and plan ahead so they can make a healthier choice Help participants develop skills to better deal with risky situations through a role playing exercise Help participants evaluate their willingness and confidence to change their behavior
3	***Substance use: weighing the pros and cons*** Provide normative feedback about alcohol use (e.g., frequency, quantity) Help participants weigh the pros and cons of substance use (including its effects on sexual behavior) so they can make thoughtful choices about their use Help participants develop strategies for decreasing negative consequences of use, as well as reaping the positive benefits without using Help participants evaluate their willingness and confidence to change their behavior
4	***Substance use: what can happen*** Provide information on the brain, and how alcohol and drug use can affect brain functioning Discuss how alcohol and drug use can affect behavior, providing personalized feedback on negative alcohol consequences as concrete examples Help participants develop strategies for protecting themselves while drinking in order to avoid negative consequences from use

AWARE incorporates education, skills building, and personalized feedback. It includes components that have been utilized in effective programs for young people experiencing homelessness [11, 51], as well as our own intervention work with other at-risk youth populations [47, 52–55]. These components include: 1. Enhancing protective factors, reducing risk factors [56–58]; 2. Targeting multiple behaviors, which is important given that targeting AOD use or sexual risk behavior in isolation does not appear to have a crossover effect on the other behavior [59]; 3. Utilizing interactive techniques that allow for active involvement in learning [54, 60–63]; 4. Reinforcing skills [64–67]; and 5. Providing information in a non-judgmental and non-confrontational manner [55, 68–71], which is important for young people experiencing homelessness who often report negative experiences with adults in “helping” roles (e.g., police, case managers, therapists, foster parents) [3, 35] and limited use of available services when they perceive staff to be judgmental [72]. These young people are often distrustful of adults offering assistance, and resistant to messages that appear to challenge their autonomy, so they may be more receptive to programs that are delivered using an MI approach [73]. Indeed, feedback from young people experiencing homelessness indicates that MI-delivered interventions are well-received by this population [1, 36, 74]. The AWARE protocol utilizes MI strategies (e.g., rulers, decisional balance) and is based upon our team’s efficacious group MI work with other populations [47, 48, 54, 68, 71, 75–77]. AWARE also combines the advantages of using a small group format to deliver the program, which allows the facilitator to capitalize on prosocial processes (e.g., reinforcement for behavior change, norm change, and vicarious learning experiences), with the provision of personalized feedback at each group session (as described more below).

**Fig. 4:**
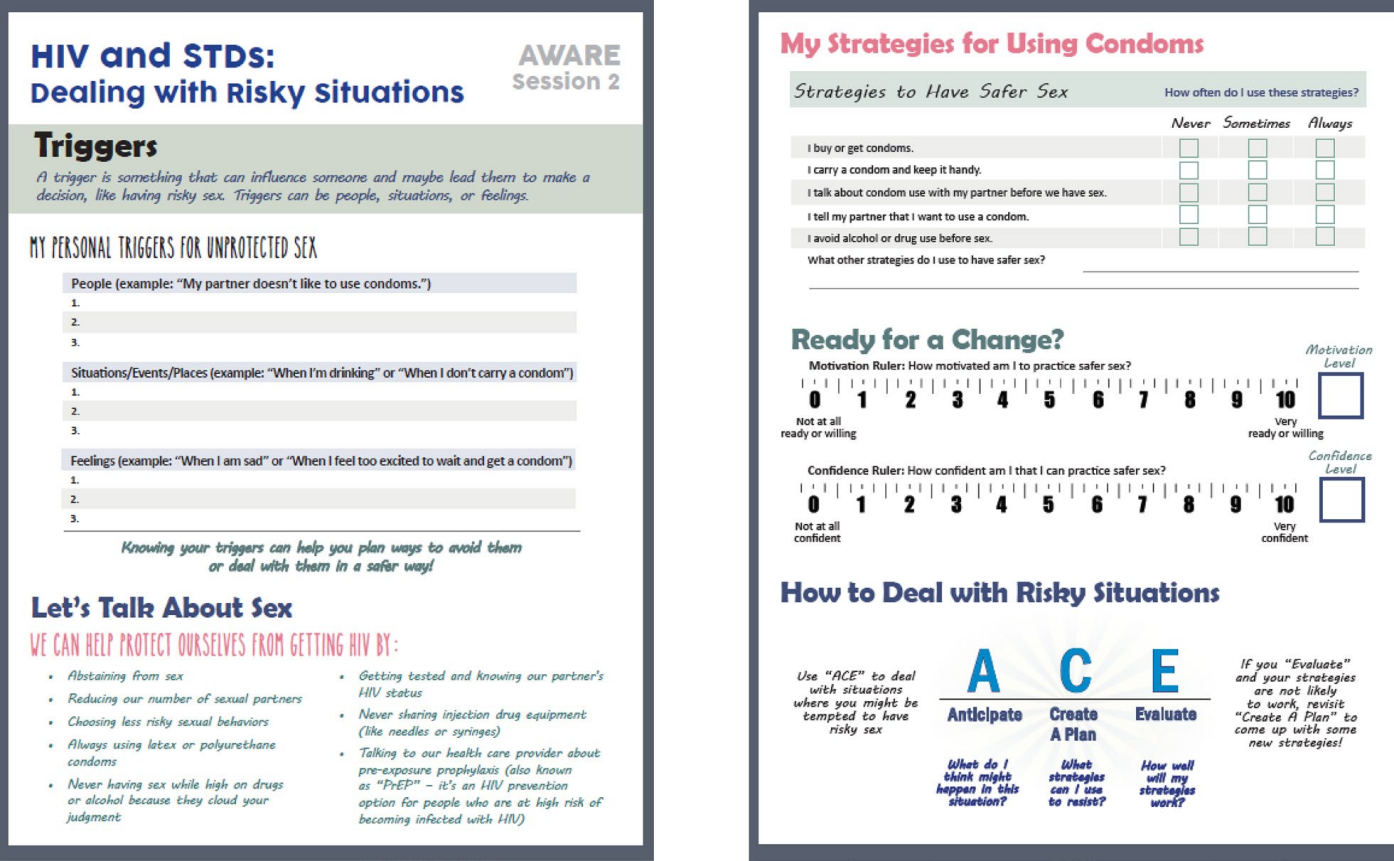
Example of AWARE feedback form

A unique feature of AWARE is that participants receive personalized feedback at each session that specifically addresses a topic being discussed during that particular session (see Fig. 4). Providing participants with personalized feedback serves a number of goals: 1. strengthening their engagement in the program by increasing its personal relevance; 2. helping them better assess their current situation and identify potential changes they can make to be safer in the future; 3. providing important information for group discussion (and leaving more time for discussion and skills building exercises since this information is collected ahead of time); and 4. providing them with additional information, such as skills training tips for making healthier choices and additional resources that are available to them both online and at the drop-in center. At each session, participants are provided with a handout with general information on AOD use and sexual risk behavior that they may find useful (e.g., links to relevant websites; how to make condom use fun), as well as fill-in information (e.g., an HIV knowledge quiz; checklist of negative alcohol consequences they have experienced recently, their triggers for unprotected sex) that is then incorporated into the group discussion. They are not required to discuss their personal experiences, and their responses on these forms are not shared with others in the group. Participants can take their feedback sheet with them after each session. Table 2 summarizes the information used to create the forms during each session.

**Table 2:** Description of session handouts

Session	Input	Output
1	HIV knowledge quiz Number of sex partners in past 3 months and number of drinks before last sexual event, and perceptions of these behaviors by peers of same age and gender	Participant’s response for each item and the correct answer Normative feedback on number of sex partners and drinking before sex, based on data from a probability sample of young adults in Los Angeles experiencing homelessness
2	Triggers for unprotected sex Sex protective strategies Motivation to change rulers	List of people, situations, and feelings that are their personal triggers for engaging in unprotected sex How often they use each strategy Separate motivation rulers showing their level of motivation to practice safe sex and confidence that they can practice safe sex
3	Participant’s past month frequency and quantity of alcohol use, and perceptions of these behaviors by peers of same age and gender Decisional balance for substance use Motivation to change rulers	Normative feedback on frequency and quantity of alcohol use, based on data from the National Survey on Drug Use and Health (NSDUH) List of personal pros and cons of AOD use, with prompts to think about (a) how to get “pros” without AOD use; and (b) how to prevent “cons” from happening Separate motivation rulers showing their level of motivation to change their AOD use and confidence that they can change their AOD use
4	Alcohol consequences survey Drinking protective strategies	Which consequences the participant has experienced recently from drinking How often they use each strategy

### Analytic plan

Baseline data will be collected from participants using self-administered paper–pencil surveys, with the survey response forms then scanned and checked for accuracy. Follow-up data will primarily be collected using either scannable paper–pencil surveys or a web-based version of the survey. Participants who cannot complete a follow-up survey in one of these ways will be offered the option of completing the survey by phone (we expect these cases to be rare). Preliminary analyses will be conducted to: (a) examine the distribution of items and correct where necessary using, for example, a log transformation for skewed outcomes and/or centering of items as appropriate; and (b) determine whether the intervention and control groups differ on baseline characteristics (t-tests and Chi square tests). Observed differences at *p* < 0.20 may be subsequently controlled for in analyses testing intervention effects. All analyses will be intention to treat–that is, we will analyze people in the groups to which they were assigned, regardless of their attendance at the sessions. In terms of missing data and attrition, our general modeling approach will allow for the inclusion of all study participants who completed the baseline survey. We will explore patterns of missingness and use logistic regression models to gain a better understanding of the baseline predictors of study drop out (i.e., non-completion of the 3-, 6-, and 12-month assessments). To accommodate missingness in analyses we will use either an inverse probability weighting approach or multiple imputation [81].

### Main outcomes of the intervention

The main outcome variables are AOD use and sexual risk behavior at 3-, 6-, and 12-month follow-up. Using items based on the Monitoring the Future survey [82], we will assess the number of days, in the past 30 days, participants engaged in the use of various substances, including alcohol use, heavy alcohol use, cannabis use, non-medical prescription drug use, and use of several types of illicit drugs (e.g., heroin, methamphetamine, hallucinogens). Information is also collected on the quantity of alcohol use (drinks per day) and marijuana use (times per day) on the days these substances are used. We are administering the Global Appraisal of Individual Needs–Short Screener (GAIN-SS) [83] to explore whether intervention effects on AOD outcomes might be weaker for those with a high probability of a past year substance use disorder (scores of 3 or greater), compared to those with a low-moderate probability of a disorder. In addition, we assess the importance of cutting down or stopping their alcohol, marijuana, and other drug use (or, if they do not currently use, the importance of remaining a non-user) with separate motivation to change rulers for each type of substance (0 = *not at all important* to 10 = *extremely important*) [84]. We are assessing sexual risk behavior using both past 90 day and past 30 day timeframes, with the main outcome being the proportion of unprotected sexual events. In addition, we are assessing condom use self-efficacy using the Self-Efficacy Instrument for Protective Sexual Behaviors [85].

### Secondary outcomes of the intervention

Secondary outcome variables for these analyses will include measures of health-related quality of life and stability. Mental functioning will be as assessed with the eight-item Patient Health Questionnaire (PHQ-8) [87] for symptoms of depression in the past 2 weeks (e.g., feeling down, depressed, or hopeless; 0 = *not at all* to 4 = *nearly every day*). Physical functioning will be assessed with items from the Physical Health Questionnaire [88], which focus on a variety of physical symptoms (e.g., difficulty sleeping, headache; 1 = *not bothered at all* to 3 = *bothered a lot*), as well as the widely used general health status item from the SF-12 (1 = *poor* to 5 = *excellent*) [89]. Social functioning will be assessed in terms of friend support in the past 30 days, using items from the PROMIS Pediatrics Peer Relationship Scale (e.g., I was able to count on my friends; 1 = *never* to 5 = *almost always*) [90], and overall satisfaction with friends (single item: 1 = *not at all satisfied* to 5 = *extremely satisfied*). Education variables include current educational attainment (1 = *no high school degree* to 5 = *college graduate*) and educational aspirations (1 = *I do not intend to receive a high school diploma or GED* to 5 = *graduate school degree*). Employment variables include current employment status, number of different paid jobs in the past 3 months, and longest time spent in any one job in the past 3 months [91]. Economic stability is assessed in terms of income in the past 30 days from various sources (e.g., job, relatives or friends, government assistance, street economy) [92], and items asking how much of a problem it was in the past 3 months to get clothes, medical care/treatment, a place to clean up, a clean and safe place to sleep, and enough to eat, (0 = *none* to 4 = *a great deal*) [93, 94]. Housing stability is assessed with items adapted from Tsemberis et al. (2007) [95], asking how often they had spent the night in various locations in the past 3 months (e.g., their own house, apartment or room; someone else’s apartment or house; emergency shelter; transitional housing program; outdoors, the street, or a park; car or other private vehicle; abandoned building; hotel/motel; 0 = *never* to 7 = *everyday*).

### Analytic plan

We will compare the intervention and control groups on each of the primary and secondary outcomes with a multilevel modeling approach using SAS PROC MIXED for continuous outcomes (e.g., past month quantity-frequency of alcohol use; proportion of protected sex acts; past month income) and PROC GLIMMIX for categorical or count outcomes with overdispersion and/or zero-inflation accounted for as needed in the modeling approach (e.g., any past month heavy drinking; number of sex partners; number of jobs in past 3 months) using restricted maximum likelihood estimation. Outcomes will be tested at each time point (3-, 6-, and 12-months) and longitudinal analyses will be used to test for differences in change over the 12-month period. Although testing at each time point provides estimates of the intervention effect at that time, the longitudinal analysis will capture patterns of change over time, that is, whether the average change on outcomes within each group produce parallel lines over time vs. lines with different slopes vs. non-linear patterns. We will explore dose–response relationships given that participants voluntarily choose to attend 1 to 4 program sessions. Although we consider any attendance as receiving the intervention, attending more sessions may be associated with better outcomes [86]. In addition, we will conduct an analysis to explore whether observed program effects differ by gender or substance use severity (defined by a score of 3 or higher on the GAIN-SS) [83] by estimating separate models for each group and/or by including an interaction term in the models.

## Limitations and alternative methods considered

The AWARE curriculum does not include specific references to marijuana (although it is discussed if a participant brings it up during a session), due to focus group feedback indicating that these young adults are not interested in attending a program that addresses their marijuana use. Nonetheless, our pilot work found positive intervention effects on motivation to change marijuana use among lower severity users [1], and we plan to examine intervention effects on marijuana use and related attitudes in this larger evaluation. The study is also limited by its exclusively reliance on self-report data, although this is typical for most research of this nature. Finally, although the evaluation is being conducted in agencies located in two distinct areas of Los Angeles County, it is unclear whether results will generalize to other agencies located in other geographic regions.

We initially considered a design in which individuals would be randomly assigned to condition within agency. However, we decided against this design given concerns on the part of the research team and drop-in center staff about implementation challenges (e.g., clients perceiving that they were being refused services that other clients were getting) and the strong potential for contamination across conditions. A group-randomized design was a much better option for this evaluation in that it addressed these concerns. We are maximizing the comparability of the intervention and control groups by having each drop-in center serve as both intervention and control site on an alternating basis, as well as using the same procedures at each drop-in center to identify and recruit participants for the study. In addition, once data are collected, we will conduct preliminary analyses to determine whether the intervention and control groups differ on baseline characteristics. This step is necessary despite randomization as randomization is not occurring at the individual level. In the pilot evaluation, which used the same procedure, there were no significant baseline group differences on demographic characteristics. However, any observed differences will be controlled for in all analyses testing intervention effects with the addition of model covariates. If we observe considerable differences in the intervention and control groups that cannot be adequately accounted for with the addition of model covariates, we will develop analytic weights using propensity methods to balance the groups. We will also control for any within-agency effects by including agency as a fixed effect (dummy coded) within the models.

## Discussion

AWARE is a brief MI intervention designed to reduce AOD use and sexual risk behavior among young adults experiencing homelessness. The prior AWARE pilot study found short-term effects on AOD use and sexual risk behavior [1], and this full-scale clinical trial provides a critical next step by evaluating its effectiveness over a 12-month period in a larger and more diverse sample. It will also examine whether AWARE, by reducing AOD use, has positive effects on secondary outcomes such as health-related quality of life and social stability. This is important as this population faces numerous challenges in the areas of mental and physical health, establishing and maintaining positive supportive connections, as well as finishing their education, securing employment, and transitioning to stable housing. There is a critical need for risk reduction programs for young people experiencing homelessness that are effective, acceptable and feasible to both participants and service providers.

## Data Availability

Once collected, deidentified data from this study will be available from the corresponding author on reasonable request 1 year after all aims of the project are completed. Requestors of data will be asked to complete a data-sharing agreement that provides for 1. a commitment to using the data only for research purposes and not to identify any individual participant; 2. a commitment to securing the data using appropriate computer technology; and 3. a commitment to destroying or returning the data after analyses are completed.

## Abbreviations

AOD: Alcohol and other drug
HIV: Human immunodeficiency virus
MI: Motivational interviewing
STD: Sexually transmitted disease
